# Acute Kidney Injury Secondary to Necrotizing Sarcoid Granulomatosis

**DOI:** 10.1155/2019/3736495

**Published:** 2019-11-05

**Authors:** Varun Mamidi, Manikantan Shekar, Jaiju James Chakola, Vamsi Krishna Makkena, Jayakumar Matcha

**Affiliations:** Department of Nephrology, Sri Ramachandra Institute of Higher Education and Research, Chennai, India

## Abstract

**Background:**

Sarcoidosis is a chronic disease characterized by noncaseating lesions involving any organ and tissue in the body. Hypercalcemia and acute kidney injury is a common renal presentation of sarcoidosis. Necrotizing sarcoid granulomatosis (NSG) is a granulomatous disease entity which presents with nodular masses of sarcoid like granuloma which primarily effects the lungs. It is a rare necrotizing variant of sarcoidosis. Extra pulmonary presentation of NSG is very rare.

**Case presentation:**

We present a 36-year-old female with hypercalcemia and acute kidney injury refractory to treatment. Whole body Flourine-18-fluorodeoxyglucose positron emission tomography computed tomography (18F-FDG PET/CT) showed increased metabolic uptake with ill-defined lesions in the liver, spleen, and pelvic lymph nodes. Biopsy of the ill-defined lesions in the liver showed necrotizing granulomatous lesions without angiitis. All the markers of tuberculosis were negative and angiotensin converting enzyme levels were elevated. Patient improved with 1 mg/kg/day oral steroid therapy and is on regular follow-up with minimal dose of steroids.

**Conclusion:**

Necrotizing sarcoid granulomatosis (NSG) is a rare systemic granulomatous disease. Due to its rarity and diagnostic difficulty, treatment is challenging for clinicians, pathologists and radiologists. Treatment of choice for symptomatic patients is steroid therapy. Prognosis is good with complete recovery.

## 1. Introduction 

Renal manifestations in sarcoidosis includes acute interstitial nephritis, altered calcium metabolism, and rarely caused by glomerular disease and obstructive uropathy. The prevalence of hypercalcemia in sarcoidosis is variable with an accepted range of 10−20% and clinically has a wide spectrum of presentations [1]. Hypercalcemia is defined as serum calcium concentration more than two standard deviations above the mean values found in individuals with normal calcium levels [2]. The two most common causes of hypercalcemia seen in about 90% of all cases are malignancy and primary hyperparathyroidism. The remaining 10% incidence is due to other disorders such as familial hypocalciuric hypercalcemia, tertiary hyperparathyroidism, hyperthyroidism, drug effects, sarcoidosis, tuberculosis, and immobilization [3]. Sarcoidosis, characterized by noncaseating granulomas, can affect any organ and tissue in the body and is a chronic multisystem disorder. Necrotizing sarcoid granulomatosis (NSG) is a granulomatous disease entity which presents with nodular masses of sarcoid like granuloma with necrosis and granulomatous angiitis which primarily effects the lungs with rare extra pulmonary involvement. Approximately, only 200 cases of NSG have been reported after its first description [4]. Here, we report a case of a middle age female presenting with hypercalcemia causing acute kidney injury secondary to this rare variant of granulomatous disease.

## 2. Case Presentation 

A 36-year-old female with history of diabetes and hypertension presented with complaints of pain in the abdomen and fatigue for a duration of one month. She denied history of vomiting, diarrhoea, fever, chills, rashes, haematuria, cough, weight loss, chest pain, and shortness of breath. On examination, vitals were stable. General and systemic examination was also normal.

Laboratory investigations revealed the total serum calcium of 13.2 mg/dl, with corrected calcium of 14.4 mg/dl and a low intact parathyroid hormone level (IPTH) of 6.8 pg/dl, and serum creatinine of 2.4 mg/dl. She denied to a history of calcium supplements intake. The serum vitamin D3 (125OH) levels are 37.6 ng/ml and vitamin D (25OH) levels are 9.9 ng/ml. The PTHrP levels could not be tested due to unavailability of the test. Work up for multiple myeloma, thyrotoxicosis, and tumor markers were negative.  Table 1 shows the relevant laboratory investigations performed. Ultrasound of the abdomen showed increased echotexture of liver, normal kidney, and no other abnormalities. The patient was treated conservatively for hypercalcemia, hyperuricemia, and renal failure.

**Figure 1:**
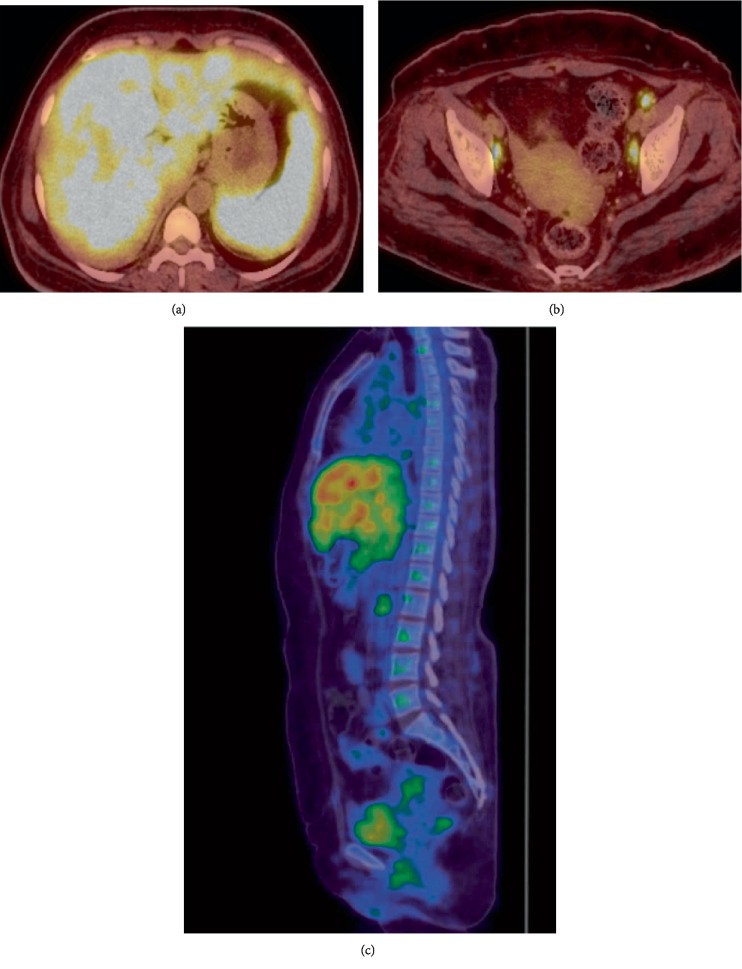
Whole body Flourine-18-fluorodeoxyglucose positron emission tomography/computed tomography (18F-FDG PET/CT). (a) Coronal PET/CT abdomen showing enlarged liver and spleen with multiple ill-defined FDG avid lesions. (b) Coronal PET/CT abdomen showing FDG avidity in few small pelvic lymph nodes. (c) Whole body sagittal PET/CT showing FDG avidity in the liver, spleen and pelvic lymph nodes.

**Figure 2:**
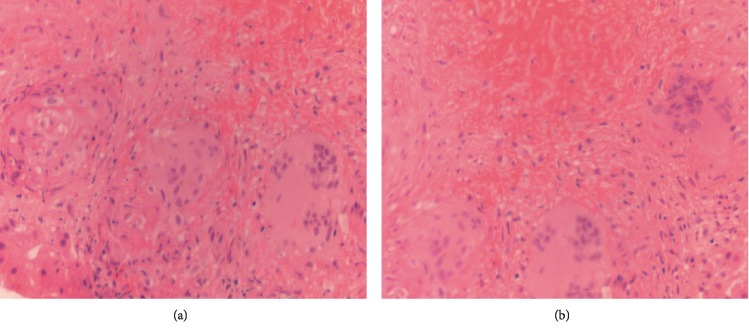
Histopathology findings. (a and b) Liver biopsy showing multifocal nodular granulomatous inflammation with central necrosis and giant cells.

Since hypercalcemia and renal failure persisted even after treatment with intravenous fluids, diuretics, and calcitonin, a whole body flourine-18-fluorodeoxyglucose positron emission tomography computed tomography (18F-FDG PET/CT) was done which showed lesions with increased metabolic activity in the liver, spleen, and left internal iliac, left external iliac, right internal iliac lymph nodes as shown in Figure 1. Hence, CT—guided percutaneous biopsy of the liver lesions was done which showed necrotizing granulomatous inflammation with no features suggestive of vasculitis as shown in Figure 2. 

Biopsy stain for fungi, AFB, TB PCR and blood TB QuantiFERON gold test was negative, but angiotensin converting enzyme (ACE) levels were elevated (119 mcl, Normal range: 8−53 mcl).

The patient was diagnosed with sarcoidosis of liver with necrotizing granulomatous lesions which is a rare variant of sarcoidosis. The patient was started on 1 mg/kg/day prednisolone. After one month follow up the patient was symptomatically better with normal calcium levels (8.6 mg/dl), reduced ACE levels (<50 mcl) and with serum creatinine of 0.9 mg/dl (eGFR- 82 ml/min/1.73m2). Steroids were tapered after one month and presently she is on a regular follow up with minimal dose of steroids.

## 3. Discussion

Granulomatous disease can either be due to infectious or a noninfectious aetiology and it commonly affects the lungs. Infectious aetiology can be due to tuberculosis, nontuberculous mycobacteria, fungi, some bacteria, and parasites (dirofilaria). Noninfectious causes include sarcoidosis, hypersensitivity pneumonitis, berylliosis, aspiration pneumonia, Wegener's granulomatosis, churg-strauss syndrome or other systemic diseases [5]. Well-formed nonnecrotizing granulomas are seen in sarcoidosis, whereas presence of necrosis signifies infection or Wegener granulomatosis [6].

Necrotizing sarcoid granuloma (NSG) is a rare controversial entity showing features that lie in between sarcoidosis and Wegener granulomatosis. These are sarcoid like granulomas with extensive necrosis and vasculitis. It was first reported by Dr. Averill A. Liebow in a series of 11 patients in 1973 [7]. He speculated that NSG may be a form of angiocentric granulomatosis showing a far better prognosis, in contrast to the other forms, or that it may be a variant of sarcoidosis [6].

There are around 200 reported cases of NSG in the last 43 years [4]. There are only 3 case reports of extrapulmonary NSG involving the liver [8–10] and only one case report with NSG liver presenting with hypercalcemia [8]. According to Karpathiou et al., median age at diagnosis was 42 years with female predominance. Clinical manifestations in NSG are mild compared to other necrotizing granulomatous diseases such as tuberculosis. The usual clinical manifestations are cough, fever, dyspnoea, weight loss, night sweats, and fatigue. About 40% can be asymptomatic at presentation. Usually it affects the lungs, the subcutaneous tissue of the skin, the kidney, the gastrointestinal tract, the liver, and the spinal cord may also be involved. Extrapulmonary manifestations are seen in 30% of the patients and involvement of the liver is very rare. Histologically, it shows a triad of sarcoid granulomas, vasculitis, and large areas of necrosis. On imaging a solitary mass, hyperfixating in PET scan is often seen. Differential diagnosis includes nodular sarcoidosis, Wegener's granulomatosis, and tuberculosis [6]. In our case, it was a 36-year-old female with pain in the abdomen and fatigue. Patient had extra pulmonary involvement with NSG in the liver diagnosed by 18F-FDG PET-CT and histology.

The key histopathologic features of NSG includes vasculitis, granuloma with necrosis, and giant cell infiltrations [4]. Very rarely it may lack these features [6]. In the histopathology of the present case vasculitis was not apparent. However, features such as biopsy of liver showing granulomas with necrosis and giant cells, hypercalcemia, elevated ACE levels, response to steroids, negative workup for other causes for hypercalcemia, and renal failure clench the diagnosis of NGS in this case.

Mechanism of hypercalcemia in sarcoidosis is due to increased 1*α* -hydroxylase activity in the alveolar macrophages. Hence there is excessive calcitriol synthesis resulting in increased intestinal calcium absorption. Hypercalcemia is rare in extra pulmonary involvement. Our patient had no pulmonary involvement and had low 25-OH vitamin D levels and normal calcitriol levels with possibility suggesting increased calcitriol production in extra pulmonary granulomatous tissue such as the liver. Possibility of occult pulmonary disease should be considered even though work up for pulmonary involvement was negative [8].

Regardless of the aetiology, the clinical features of hypercalcemia are the same. Hypercalcemia can present with symptoms involving many organ systems such as dehydration, nausea, vomiting, abdominal pain, and mental confusion. Several mechanisms involved with acute kidney injury in the context of hypercalcemia include prerenal involvement, direct alterations in vascular tone, and glomerular permeability. Due to this direct vasoconstrictive effect of calcium the renal consequences that occur are: arteriolar vasoconstriction, reduction in glomerular ultrafiltration coefficient (Kf), reduction in tubular sodium reabsorption, nephrogenic diabetes insipidus, prerenal azotaemia, acute tubular necrosis, nephrocalcinosis, and tubulointerstitial fibrosis [11]. This intrarenal vasoconstriction is also one of the mechanism of renal toxicity in rhabdomyolysis [12]. In the present case both prerenal involvement or direct vasoconstrictive effect of the calcium ion on vascular smooth muscle can be the probable mechanism of AKI secondary to hypercalcemia.

According to literature ACE level is used to monitor the disease activity in sarcoidosis. With hypercalcemia as a major clinical manifestation, calcitriol level may be useful in guiding therapy. According to a case report by Priyadarshini B et al., patient presenting with hypercalcemia as major clinical manifestation and has persistently elevated ACE level, calcitriol level monitoring can help guide steroid dose and provide the potential to prevent symptomatic hypercalcemia. In acute setting calcitriol level monitoring will help to guide steroid therapy whereas ACE levels may be useful for chronic disease monitoring rather than treatment guidance when sarcoidosis is associated with hypercalcemia [8].

Corticosteroids are mainstay of treatment. Prednisone (1 mg/kg/day) tapered over several weeks to months is recommended. In steroid resistant cases azathioprine, methotrexate, and hydroxychloroquine may be used (Table 2). There are no gold standard tests to monitor therapy. Over all prognosis for NSG is good and depends on gender, race, age, organ involvement, and presenting clinical manifestations.

## 4. Conclusion

Necrotizing sarcoid granulomatosis (NSG) is a rare systemic granulomatous disease. Histologically NSG is defined by sarcoid-like granulomas, granulomatous vasculitis and necrosis superimposed on confluent granulomas [13]. Clinical presentation is nonspecific. Due to its rarity and diagnostic difficulty, treatment is challenging for clinicians, pathologists, and radiologists (Table 2). Treatment of choice is steroids. Prognosis is good, but relapse is common.

## Figures and Tables

**Table 1:** Laboratory values.

Test name	Result	Normal range
Hemoglobin (gm/dl)	9.8	12.0–15.0
Total count (cells/cumm)	10800	4000–11000
Polymorphs	78.6%	45%–70%
Lymphocytes	8.3%	25% –40%
Eosinophils	3.7%	1%–6%
Basophils	0.2%	0%–1%
Platelet count (lakhs/cumm)	4.35	1.5–4.5
Blood urea nitrogen (mg/dl)	22	5–21
Serum creatinine (mg/dl)	2.4	0.60–1.10
Serum sodium (mmol/l)	134	136–146
Serum potassium (mmol/l)	3.6	3.5–5.1
Serum chloride (mmol/l)	98	101–109
Serum bicarbonate (mmol/l)	26	21–31
Serum calcium (mg/dl)	13.2	8.8–10.6
Serum phosphorus (mg/dl)	4.0	2.5–4.5
Serum uric acid (mg/dl)	11.1	2.6–6.0
Serum intact parathyroid hormone (IPTH) (pg/ml)	6.8	12–88
Serum vitamin D (25OH) (ng/ml)	9.9	<20
Serum vitamin D3 (125OH) (ng/ml)	37.6	19.6–54.3
Random plasma glucose (mg/dl)	109	70–140
Urine colour	Straw yellow/clear	
Urine pH	6.0	4.6–8
Urine specific gravity	1.005	1.001–1.035
Urine glucose	Negative	Negative
Urine protein	Negative	Negative
Urine bilirubin	Negative	Negative
Urine ketone	Negative	Negative
Urine urobilinogen (Eu/dl)	0.2	0.2–1.0
Urine pus cells (cells/hpf)	2–3	<5 cells
Urine RBC (cells/hpf)	Nil	0–2
Urine epithelial cells (cells/hpf)	2–3	0–4
Urine casts (cells/hpf)	Nil	Nil
Urine crystals (cells/hpf)	Nil	Nil
Urine protein creatinine ratio (mg/mmol)	0.90	<0.3
24 h Urine calcium (mg/day)	160	<250 mg/day
Urine bence jones protein	Negative	Negative
Total bilirubin (mg/dl)	0.67	0.3–1.2
Direct bilirubin (mg/dl)	0.20	<0.2
SGOT (U/L)	19	<35
SGPT (U/L)	16	<35
Total protein (gm/dl)	6.8	6.6–8.3
Albumin (gm/dl)	2.8	3.5–5.2
Globulin (gm/dl)	4.0	2.0–3.5
Alkaline phosphatase (U/L)	286	30–120
ANA	Negative	Negative
C3 (mg/dl)	164.0	90–180
C4 (mg/dl)	55.70	10–40
PR3–ANCA by ELISA	<1:40	<1:40–Negative
MPO – ANCA by ELISA	<1:40	<1:40–Negative
FT3 (pmol/l)	2.92	2.5–3.9
FT4 pmol/l)	1.38	0.56–1.50
TSH (mU/L)	1.660	0.34–5.60
Carcino embryonic antigen (CEA) (mcg/l)	1.3	<3.0
Alpha feto protein (mcg/l)	1.15	<0.9
Angiotensin converting enzyme levels (ACE) (nmol/ml)	119	
HIV	Nonreactive	Nonreactive
HBSAG	Nonreactive	Nonreactive
HCV	Nonreactive	Nonreactive
Peripheral smear	Normocytic Normochromic Anemia	

**Table 2:** Teaching points.

(1) Necrotizing sarcoid granuloma (NSG) is a rare controversial entity showing features that lie in between sarcoidosis and Wegener granulomatosis with sarcoid like granulomas and extensive necrosis.
(2) The usual clinical manifestations are cough, fever, dyspnea, weight loss, night sweats, fatigue. About 40% can be asymptomatic at presentation. Extra pulmonary manifestations are seen in 30% of the patients and involvement of liver is very rare.
(3) Histologically, it shows a triad of sarcoid granulomas, vasculitis and large areas of necrosis. On Imaging a solitary mass hyperfixating in PET Scan is often seen. Differential diagnosis includes nodular sarcoidosis, Wegener's granulomatosis and tuberculosis.
(4) Corticosteroids are mainstay of treatment. Prednisone (1 mg/kg/day) tapered over several weeks to months is recommended. In steroid resistant cases azathioprine, methotrexate and hydroxychloroquine may be used.
(5) Due to its rarity and diagnostic difficulty, treatment is challenging for clinicians, pathologists and radiologists. Prognosis is good, but relapse is common.

## Abbreviations

NSG: Necrotizing sarcoid granulomatosis
18F-FDG PET/CT: Flourine-18-fluorodeoxyglucose positron emission tomography computed tomography
IPTH: Intact parathyroid hormone level
AFB: Acid fast bacilli test
TB: Tuberculosis
TB PCR: Tuberculous polymerase chain reaction
ACE: Angiotensin converting enzyme
eGFR: Estimated glomerular filtration rate.
